# Injectable Artificial Photoreceptors: STEM Cell Functional Integration and in vivo Electrophysiological Validation in Retinal Degeneration Models

**DOI:** 10.1002/smsc.70353

**Published:** 2026-07-31

**Authors:** Afshin Izadian, Ali Torkashvand, Benjamin Allan McCall, Yong Gao, Christine M. Taylor, Fateme Karimi Hafshejani, Arupratan Das, Michelle L. Surma, Sunland Gong, Amir Reza Hajrasouliha

**Affiliations:** ^1^ Electrical Engineering Technology Purdue University West Lafayette Indiana USA; ^2^ Purdue Institute for Integrative Neuroscience West Lafayette Indiana USA; ^3^ Eugene and Marilyn Glick Eye Institute Indiana University School of Medicine Indianapolis Indiana USA

**Keywords:** artificial photoreceptors, intravitreal injection, pupillary light reflex, retina ganglion cells, retina prosthetics, visual evoked potential

## Abstract

In this study, an injectable artificial photoreceptor (APR) is engineered to transduce visible light into cell‐level electrical stimulation via localized surface plasmon resonance to restore vision in patients with vision loss. APRs comprise gold nanoparticles (AuNPs) with poly (vinylidene fluoride‐co‐hexafluoropropylene) (PVDF‐HFP) dielectric coating and are integrated onto a high‐permittivity barium titanate core. This yields a stable nanocomposite with maximum absorbance in the green band at around 525 nm. APRs were evaluated in vitro using human pluripotent stem cell‐derived retinal ganglion cells (hRGCs) exposed to light stimulation. In vivo efficacy was assessed following intravitreal injection of APRs in the *rd1* mouse model, and functional outcomes were evaluated using visual evoked potentials (VEPs) and pupillary light reflex (PLR) testing. In vitro, optical stimulation of hRGCs in the presence of APRs increased multielectrode‐array (MEA) firing. This shifted cellular metabolism toward mitochondrial oxidative phosphorylation without inducing apoptosis. In *rd1* mice, intravitreal APR delivery produced robust restoration of light‐evoked retinal activity in explant MEAs and improved functional readouts in vivo, validated by enhanced PLR and increased N1 amplitudes on flash VEPs. This study demonstrates that a fully injectable nanophotonic prosthesis can restore light responsiveness in a retinal degeneration model without the need for implanted electronics or genetic modification.

## Graphical abstract

Artificial photoreceptors are engineered to efficiently absorb light, with the strongest sensitivity in the green region of the visible spectrum. Upon illumination, they generate electrical signals that activate retinal ganglion cells, which are typically unaffected by disease, enabling transmission of visual information to the brain. This approach offers a promising strategy for future vision restoration technologies.

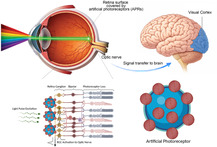

## 1 Introduction

Degenerative retinal diseases, such as retinitis pigmentosa (RP) and advanced age‐related macular degeneration (AMD), culminate in the loss of rods and cones and progressive blindness [1]. Despite extensive photoreceptor degeneration, inner retinal neurons, including retinal ganglion cells (RGCs), can persist and remain electrically excitable [2]. This enables strategies that bypass the damaged outer retina [3, 4]. Current clinical and investigational approaches include epiretinal electrode arrays [5, 6], optogenetic sensitization of inner retinal cells [7], and cell replacement [8]; however, these methods face challenges such as surgical invasiveness, limited spatial resolution [9, 10], or the need for viral gene transfer [11] and high‐intensity illumination.

Nanotechnology is currently gaining attention as a promising solution for various ophthalmic diseases [12, 13, 14, 15, 16]. This fascination stems from nanoparticles’ unique size and physical properties, which can be tailored to cross cell barriers, deliver drugs effectively, or function as needed across various applications. These properties also make nanotechnology an area of interest for retinal conditions [15]. Retinal dystrophies and degenerative disorders are leading causes of photoreceptor loss and permanent visual impairment [17]. RP is the most common retinal dystrophy, affecting roughly 1 in 5000 individuals [18]. It is a progressive genetic disorder that initially affects rods and eventually cones, leading to a significant decline in visual function.

Advanced dry AMD also causes geographic atrophy, resulting in a central scotoma due to photoreceptor loss. However, in most dystrophic/degenerative retinal disorders, despite significant photoreceptor loss, the RGCs remain intact, which opens opportunities for visual‐restoring approaches [19].

The RGCs connect photoreceptor cells to the nervous system, making them a promising therapeutic target and a focus of vision research. Direct stimulation of RGCs using artificial photoreceptors (APRs) may be a solution for restoring vision for a number of retinal conditions [20].

APRs have previously been reported as complementary to enhancing gene therapy, trophic factor therapy, visual cycle inhibitors, and cell transplants [21]. Direct RGC activation has been investigated previously by implanting electrodes on the retina surface (e.g., the Argus II, artificial retina implant). Using a complex vision‐processing algorithm, patients could recognize single letters or object borders [22, 23]. However, these implants require a complex surgical technique, offer low resolution, and have limited clinical use with current technology. Therefore, there is an unmet need for the treatment of photoreceptor loss. Using localized surface plasmon resonance (LSPR) and the dielectric properties of the nanoparticles, we developed APRs that can produce a bioelectrical signal in response to visible light.

LSPR is an optical property of metallic elements in which a specific wavelength of light creates oscillations in free electrons [24]. The oscillation of free electrons creates an electromagnetic field on the particle surface, which can be used in various applications, as resonant wavelengths vary with different elements, particle sizes, and particle shapes [25]. In this study, we formulated a nanocomposite that uses the optically induced LSPR property of AuNPs to trigger RGCs. This nanocomposite was evaluated in mice and showed potential to restore vision, suggesting a possible treatment for retinal dystrophies.

## 2 Materials and Methods

All animal procedures were approved by the Institutional Animal Care and Use Committee (IACUC) at Indiana University School of Medicine (protocol numbers: IACUC 25068 MD/R/HZ and IACUC 22117; IBC Protocol #1191 and IBC Protocol #IN‐1015, updated for IACUC).

### 2.1 Design Rationale and Composition of Artificial Photoreceptors

APRs were designed as a multilayer dielectric–plasmonic system in which AuNPs serve as the core that mediates LSPR charges; PVDF‐HFP serves as a biocompatible plasmonic dielectric coating to promote capacitive coupling; and barium titanate nanoparticles (BTNPs) act as a high‐permittivity core to amplify polarization and stabilize the composite. The final formulation of APR was prepared as a colloidal suspension suitable for intravitreal injection (IVI).

### 2.2 Nanoparticle Synthesis

**FIGURE 1:**
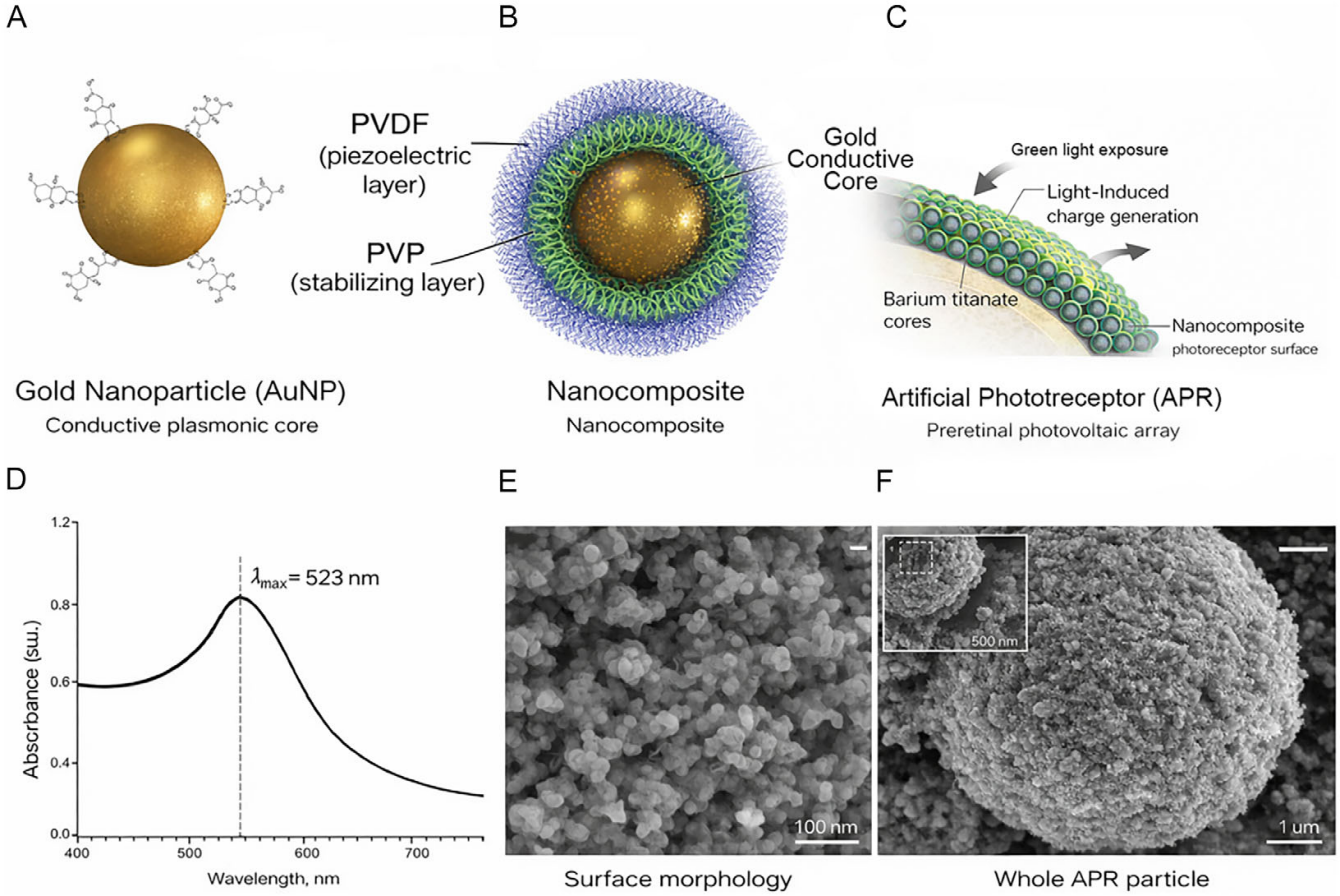
The process of forming an APR is as follows: (A) Gold nanoparticles (AuNPs) of a specific size are formed in citrate ion packaging. (B) Use of PVP to link AuNP and PVDF to form the nanocomposite (NC). (C) Packaging of NC on barium titanate (BT) cargo and to make APR. (D) Absorption profile of APRs using UV–Vis spectrophotometry. Panels (E,F) depict SEM images of the nanocomposite (NC) and APR, respectively.

Gold (III) chloride trihydrate (≥99.9% trace metals basis), trisodium citrate dihydrate, polyvinylpyrrolidone (≈10 kDa), poly (vinylidene fluoride‐co‐hexafluoropropylene), and *N*,*N*‐dimethylformamide (≥99%) were purchased from Sigma‐Aldrich Co., St. Louis, MO, USA. Barium titanate nanoparticle powder was purchased from SkySpring Nanoparticles, Inc., Houston, TX, USA. Ultrapure water was obtained using ELGA Lab Water PURELAB flex (18.2 MΩ). Gold nanoparticles (AuNPs) were synthesized as previously described [26] using a modification of the Turkevich et al. method and Frens et al. [27, 28, 29]. Briefly, gold (III) chloride trihydrate was added to boiling ultrapure water while magnetically stirred. Trisodium citrate dihydrate was then added to the gold chloride solution. The reaction solution continued to boil, turning wine‐red, before it was removed from the heat and stirred as it cooled to room temperature. Nanoparticles were sealed, stored in a cool environment, and covered to prevent light exposure. Polyvinylpyrrolidone (PVP) was added to the synthesized AuNPs before they were removed from the heat. This replaced the citrate coating of the nanoparticles, serving as a linker molecule between AuNPs and another polymer. Poly (vinylidene fluoride‐co‐hexafluoropropylene) (PVDF‐HFP) is a dielectric polymer that coats the PVP‐AuNPs [30]. The NP solution and PVDF were separately dissolved in dimethylformamide (DMF) and then mixed to form the desired nanocomposite (NC). Barium titanate (BTO) nanoparticles (BTNPs) were used as a base core for attaching AuNPs and NC [31, 32]. BTNPs were functionalized with (3‐aminopropyl) triethoxysilane (APTES) and mixed with NC to produce the final product of the APRs. Schematic Figure 1 illustrates the development process of APRs.

Gold nanoparticles (AuNPs), PVDF‐HFP, and BaTiO_3_ (BTO) were selected because they provide complementary optical, mechanical, dielectric, and electrical functions within the nanocomposite [33, 34, 35, 36, 37]. AuNPs provide a light‐responsive component, as gold nanoparticles exhibit LSPR, strong absorption in the visible range, and chemical stability [34, 38]. PVDF‐HFP serves as the polymer matrix that combines flexibility, high dielectric constant, chemical resistance, solution processability, and electroactive behavior [33, 36]. The electroactive response of PVDF is stronger in the β‐phase polar crystalline form. BTO was incorporated as a lead‐free ferroelectric/piezoelectric ceramic to provide high dielectric permittivity and to improve the dielectric and polarization properties of polymer composites [35, 37]. In PVDF‐based composites, BTO can also promote the development of polar phases and strengthen interfacial polarization, thereby enhancing the electroactive performance of the polymer matrix [37, 39].

### 2.3 Physiochemical Characterization

Hydrodynamic size and stability were characterized using dynamic light scattering (DLS) [Zetasizer Nano ZS90, Malvern Panalytical]. APRs’ Morphology and their stability in solution were assessed through scanning electron microscopy (SEM) [JSM‐7800F Schottky Field Emission SEM, JEOL]. Ultraviolet–visible spectroscopy (UV–Vis) [BioTek Synergy H1 Multimode Reader, Agilent, Santa Clara] quantified the LSPR absorbance and confirmed a peak of APRs in the green band (520–525 nm) [40, 41].

### 2.4 Human Stem Cell Differentiation to Retinal Ganglion Cells Differentiation

Human embryonic stem cell (H7‐hESCs) reporter lines were used for human retinal ganglion cell (hRGC) differentiation. This reporter line contains a CRISPR‐engineered multicistronic BRN3B‐P2A‐tdTomato‐P2A‐Thy1.2 construct into the endogenous RGC‐specific BRN3B locus and was used for hRGC differentiation as demonstrated previously [41, 42, 43]. In brief, stem cells were dissociated with accutase and neutralized using mTeSR1 media (mT) with 5 μM blebbistatin. Cells were seeded at 100 000 per well in a 24‐well matrigel (MG) coated plate. The next day, the media was exchanged with mT, and the day after with differentiation media (iNS). Small molecule‐based differentiation was then carried out [41]. Successful hRGC differentiation was judged by the tdTomato expression, and after 45 days of differentiation, hRGCs were purified using magnetic activated cell sorting with Thy1.2 microbeads [42]. Cells were resuspended in iNS media, counted, and then seeded into MG‐coated plates for experiments.

### 2.5 Optical Stimulation Paradigm and Viability/Caspase Activity

Purified hRGCs were seeded at 25 000 cells per well of a 96‐well clear‐bottom black‐walled MG‐coated plate and maintained for 3 days. Nanoparticles were added to the medium to a final concentration of 100 ppm. The plates were then exposed to green light at 40 Hz for 24 h using the Lumos system from Axion Biosystems. During this exposure, the control wells were covered with foil to prevent light exposure. After light exposure, viability and caspase activity were measured according to the kit protocol (Promega ApoTox‐Glo Triplex Assay). The assay uses live‐cell protease activity measurements with a fluorogenic (400Ex/505Em), cell‐permeant peptide substrate to assess the live‐cell population. Subsequently, the same cultures were assessed for apoptosis by adding a second reagent that lyses cells and contains a luminescent substrate for active caspase‐3/7. Luminescence is proportional to cellular apoptosis.

### 2.6 Seahorse ATP Rate Assay and Cytotoxicity

The 96‐well Seahorse plate was coated with MG, and hRGCs were seeded at 250 000 cells per well and maintained for 2 days. Twenty‐four hours before Seahorse measurements, the medium was exchanged to iNS containing 100 ppm nanoparticles, and the Seahorse was then exposed to green light at 40 Hz for 24 h using the Lumos system from Axion Biosystems. The Seahorse ATP rate assay was performed using an XFe96 analyzer (Agilent, Santa Clara) following the previously published protocol [40]. Images of each well were taken before the assay, and the total cell area was calculated to normalize the data. The data were analyzed using Seahorse Wave Desktop software (Agilent, Santa Clara).

### 2.7 Retinal Explant Multielectrode Array

A multielectrode array (MEA; Maestro Edge, Axion Biosystems, Atlanta, GA), equipped with the Lumos multiwell light delivery system and Lumos 96‐well MEA plate (Axion Biosystems), was used to evaluate in vitro hRGCs cultured under a temperature‐ and gas‐regulated (O_2_/CO_2_) environment. Pulses of green (530 nm) and blue (475 nm) light at various intensities were used to optically trigger electrical signals from the cells, which were then detected by the MEA. The Axion Biosystems software was used to process and organize the resulting data.

The same MEA machine was used to record signals from retinal explants mounted on a custom mesh with the ganglion cell side facing the MEA electrodes. A 24‐well Lumos MEA plate (Axion BioSystems) was utilized to assess light responsiveness of the retinal explants using 25 and 40 Hz stimulation with green and blue light in Ames’ medium (Sigma‐Aldrich, St. Louis, MO, USA), under optimized O_2_/CO_2_ levels, temperature, and pH conditions. Retinal explants from *rd1* mice with established retinal degeneration (Figure S1) that had received an intravitreal APR injection 1 day earlier were compared with noninjected *rd1* and wild‐type (WT) control retinas.

For the ex vivo MEA experiments, the biological replicate was defined as an individual retinal explant. For both green and blue light stimulation, five APR‐treated *rd1* retinal explants, three WT retinal explants, and three sham‐treated *rd1* retinal explants were included. A total of 7, 7, and 13 MEA recording sessions were performed for APR‐treated *rd1*, WT, and sham‐treated *rd1* explants, respectively, during green light stimulation, and 10, 8, and 8 recording sessions, respectively, during blue light stimulation. Electrophysiological parameters were calculated using Axion Neural Metrics software. Electrode‐level recordings were summarized to generate a single value for each retinal explant before statistical analysis. Consequently, the retinal explant was considered the biological and statistical unit, whereas individual electrodes and repeated recordings were treated as technical measurements and were not analyzed as independent observations, thereby avoiding pseudoreplication. Details regarding the MEA recording protocol and parameters are provided in the Supporting Information file.

Using the measured nanoparticle absorbance spectrum (Figure 1D), with a plasmon peak at *λ*max ≈ 523 nm and absorbance values of ≈0.82 at 530 nm and 0.60 at 475 nm, the fractions of incident light removed from the beam are 1–10^−^^0.82^ ≈ 0.849 and 1–10−0.60 ≈ 0.749, respectively. Assuming, conservatively, that this attenuated light is converted into heat, a 1.9 mW optical stimulus applied for 120 s would deposit ≈0.194 J at 530 nm and 0.171 J at 475 nm. In the Axion Lumos/Maestro configuration, light is delivered from above and distributed across each well. The manufacturer reports well volumes of 500 µL for the Lumos MEA 96 plate and 2000 µL for the Lumos MEA 24 plate. Treating the culture medium or Ames’ medium as water‐like, the corresponding upperbound bulk temperature rises are therefore ≈0.093 °C (530 nm) and 0.082 °C (475 nm) for the 96‐well hRGC assay, and 0.023 °C (530 nm) and 0.021 °C (475 nm) for the 24‐well retinal explant assay.

### 2.8 Intravitreal Injection

IVIs involved injections into the vitreous of *rd1* mice (Jackson mice) with 1 µL of BTNC at 1000 µg/mL in the treatment group and the same volume of saline injection in the *rd1* control mice in both eyes. Mice were anesthetized for 1 h with 5–10 mg/kg xylazine or 0.5 mg/kg dexmedetomidine and 75 mg/kg ketamine via intraperitoneal injection. Topical antiseptic betadine (iodopovidone) eye drops were administered before an IVI of 1 µL of APRs (1000 µg/mL). After IVI, mice were placed on heating pads until they recovered from anesthesia and returned to their respective cages. The same anesthetic procedure was also done for optical coherence tomography (OCT) imaging, visual evoked potential (VEP) testing, and pupillary light reflex (PLR) testing.

### 2.9 In Vivo Functional Readouts (Visual Evoked Potential and Pupillary Light Reflex)

Flash VEPs (Celeris, Diagnosys LLC) were recorded to assess the visual cortex's electrical activity in *rd1* mice in response to light stimulation. A light‐emitting diode (LED) source of green and blue light with an intensity of 5 cd s/m^2^ and a duration of 1 ms was utilized to activate the APRs. The light stimulation was in the range of conventional electrophysiological studies [44]. Recordings were conducted before IVI and on Days 1, 3, and 7 post‐IVI. Details of VEP testing are provided in the Supporting Information file. For the VEP study, 13 eyes of 10 *rd1* mice were used for green light stimulation and eight eyes of 7 *rd1* mice were recruited for blue light optical stimulation.

### 2.10 Pupillary Light Reflex

PLR was measured in the eyes of mice to evaluate the effectiveness of APRs using an infrared handheld microscope [AM4115FIT, Dino‐Lite Edge Digital Microscope]. PLR measurements were performed at baseline (before IVI) and on Days 1 and 7 after IVI in mice that had been kept in dark adaptation conditions for at least 16 h. Infrared light from an LED source (Adafruit Super‐bright 5 mm IR LED) was directed into the eyes for 30 s (940 nm infrared LED producing an irradiance of 0.18 mW/cm2 at the corneal plane). The captured video frames were analyzed using ImageJ (National Institutes of Health, USA) by semiautomated marking of the pupillary boundary. Color images were converted to 8‐bit and binarized by the Otsu threshold method. The corneal light reflex was removed from the pupillary area using the Fill Holes function, and the pupillary area was delineated with the analyzed particle function. The segmented pupillary area was exported as an image mask. The area after exposure was subtracted from the prestimulation area, then divided by the prestimulation area and expressed as a percentage change.

### 2.11 Statistical Analysis

For nonrepeated parametric variables, analysis of variance (ANOVA) was applied. Repeated measures were analyzed using a general linear model, and if there were no missing values, a mixed‐effect model was used. Post hoc tests were implemented for pairwise comparisons with baseline or controls. Kruskal–Wallis was used for variables that are not normally distributed. Values are presented as mean ± standard error of the mean (SEM). A *p*‐value ≤ 0.05 was considered statistically significant. The analyses were done by GraphPad Prism, version 10.3.1.

Figures were created using Rhino 8 (Robert McNeel & Associates, Seattle, WA, USA), BioRender (BioRender.com), and finalized in Adobe Illustrator (version 28.7.1, Adobe Systems, San Jose, CA, USA).

## 3 Results

### 3.1 Artificial Photoreceptors Exhibit Green Band Localized Surface Plasmon Resonance and Uniform Colloidal Morphology

We developed an injectable colloidal nanocomposite solution as APRs with potential for photoreceptor loss replacement. We modified the Turkevich method to synthesize AuNPs with controlled size and minimal variation. UV–Vis spectroscopy confirmed the maximum absorption of the nanoparticles in the desirable green range from 520 to 525 nm (Figure 1D). Evaluation of the product's size and shape using SEM and DLS revealed uniform size and dispersity (Figure 1E,F).

### 3.2 Artificial Photoreceptors Enable Light‐to‐Voltage Conversion

In addition to MEA and VEP recordings, direct evidence of light‐triggered charge generation by APRs was obtained using a voltage‐sensitive dye (Di‐8‐ANEPPS) and a ChemFET‐based electrical measurement. Upon optical stimulation at 525 nm, AuNP‐based APRs induced a 635 nm Di‐8‐ANEPPS response, whereas dark controls showed no comparable signal, indicating light‐dependent charge generation by the APRs. Similarly, ChemFET measurements showed that illumination of APRs deposited on the gate region produced a light‐dependent increase in drain–source current, with a linear response of ≈9 μA/lux, demonstrating interfacial optical‐to‐electrical signal conversion. Taken together with the MEA and VEP data, these results support the conclusion that APRs generate light‐triggered interfacial charges capable of modulating neural activity.

**FIGURE 2:**
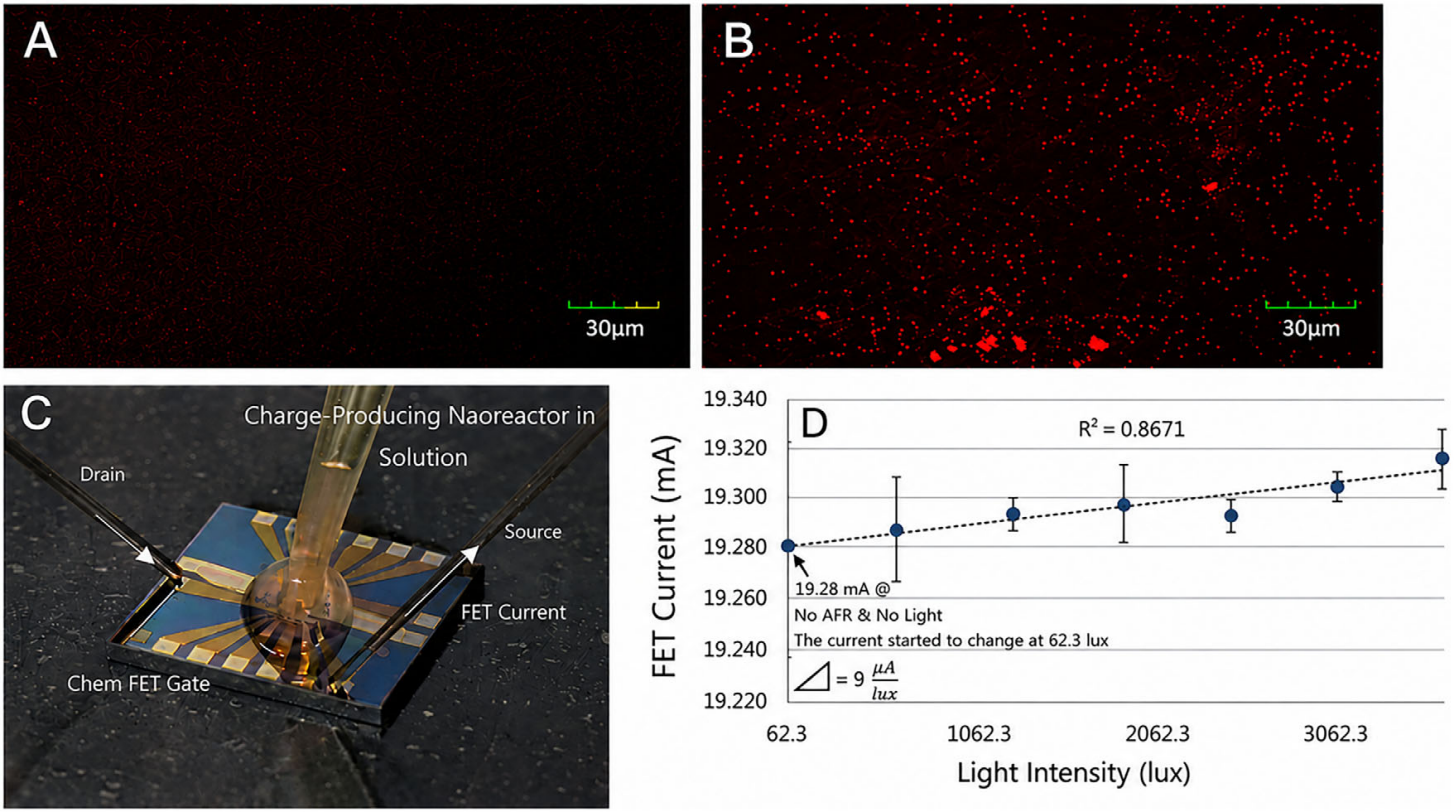
Optical excitation of APRs. (A) Control of AuNP‐based APRs, no light exposure. (B) Response of voltage dye. Red dots show the voltage generation of the AuNP‐based nanoreactor. APR Light‐to‐electric charge conversion solution. (C) ChemFET circuit schematic and the placement of APRs suspended in a droplet of DI water. An external 20 mV source was applied across the gate to establish the initial current. (D) The current increases due to the charge generated by APRs when exposed to light.

Di‐8‐ANEPPS (Thermo Fisher Scientific) was utilized to image the electric charges generated by nanoreactors. Upon excitation at 525 nm, the generated charges caused the dye to emit at 635 nm. Figure 2A,B shows a uniform distribution of APRs in solution and their ability to generate charges. Figure 2A shows the control for APR, with no response in the absence of light. Figure 2B shows the emitted light in the presence of localized voltage generation from APRs to the voltage dye.

### 3.3 The ChemFET Experiment

A 20 μL droplet of nanoreactors (100 μg/μL) was applied to cover the gate (90 × 90 μm^2^) biased with a 20 mV DC source (Figure 2C). The initial current through the ChemFET without any light exposure was 19.280 mA. Upon light exposure, the APRs began generating charges and altering the gate bias. As a result, the current flowing from port (*D*) to port (*S*) increased at a linear net rate of 9 $\frac{\mu A}{\mathrm{lux}}$ (Figure 2D). This proves that the nanoreactors could convert light exposure to ionic charges.

### 3.4 Artificial Photoreceptors Enable Light‐Evoked Spiking in Human Retinal Ganglion Cell Cultures

MEA with the light delivery system was used to excite and detect electrical signals from hRGCs in vitro in response to the green and blue light at different frequencies.

**FIGURE 3:**
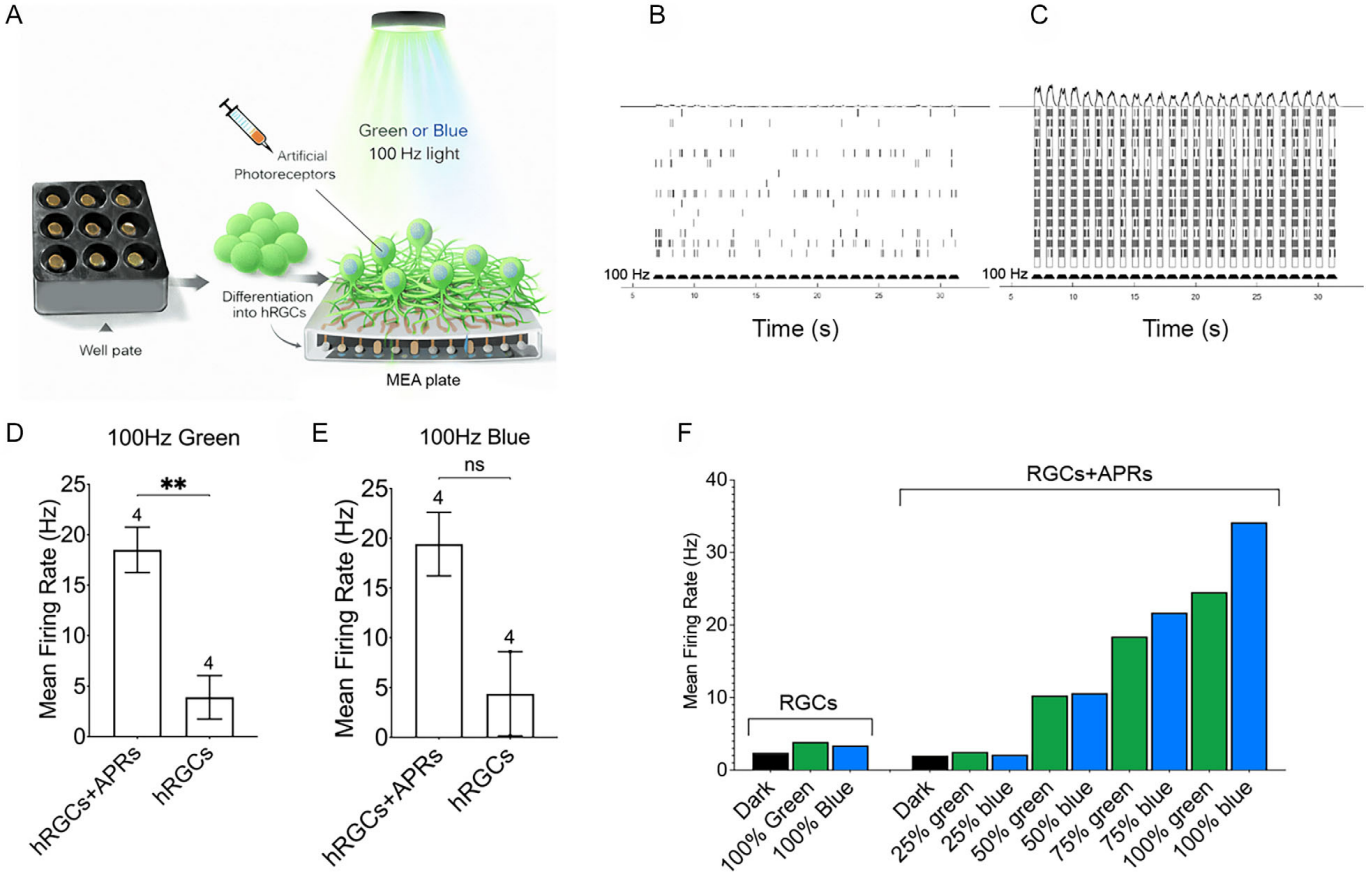
Raster plot of retinal explants in different experimental conditions (A). Comparison of electrical activity of cultured hRGCs in the absence (B) and presence of APRs (C) using 100 Hz green light stimulation. Comparison of mean firing rate between two groups using 100 Hz green (D) and blue (E) optical stimulation. Data are shown as mean ± SEM. Statistical comparisons were made using a mixed‐effects model with Dunnett's multiple comparisons test. *p < 0.05, **p < 0.01.

Our results revealed that cultured hRGCs exhibited significantly greater electrical activity when exposed to APRs in response to light stimulation compared to controls (Figure 3B–E). Furthermore, increasing light intensity increased the mean firing rate (Figure 3F).

Stem‐cell‐derived hRGCs were cultured in MEA wells to evaluate the response of hRGCs due to different light stimulation profiles (Figure 3A). The raster plot of hRGCs in the absence of APRs reveals minimal response (Figure 3B), while a significant firing of hRGCs is evident with the presence of APRs in response to 0.5 s ON/0.5 s OFF light stimulation (Figure 3C). Cultured hRGCs showed an increased firing rate in response to 100% intensity, which is statistically significant for green (Figure 3D), but not for blue light stimulation. Different light stimulation profiles are implemented to assess APRs’ functions. In the absence of APRs, hRGCs show minimal response to maximum intensity (100%) of blue and green light stimulation compared to those hRGCs that are treated with 100 ppm of APRs, which show an increasing mean firing rate (Figure 3F) by increasing light intensity. Uniformly sized gold nanoparticles within APRs resonate with a specific wavelength spectrum, and their activity increases with the increased intensity of blue or green light. In addition, our results supported the efficacy of APRs at different frequencies compared to the control group (Figure S2).

### 3.5 Optical Stimulation of Artificial Photoreceptors Drives OXPHOS Without Causing Human Retinal Ganglion Cell Toxicity

**FIGURE 4:**
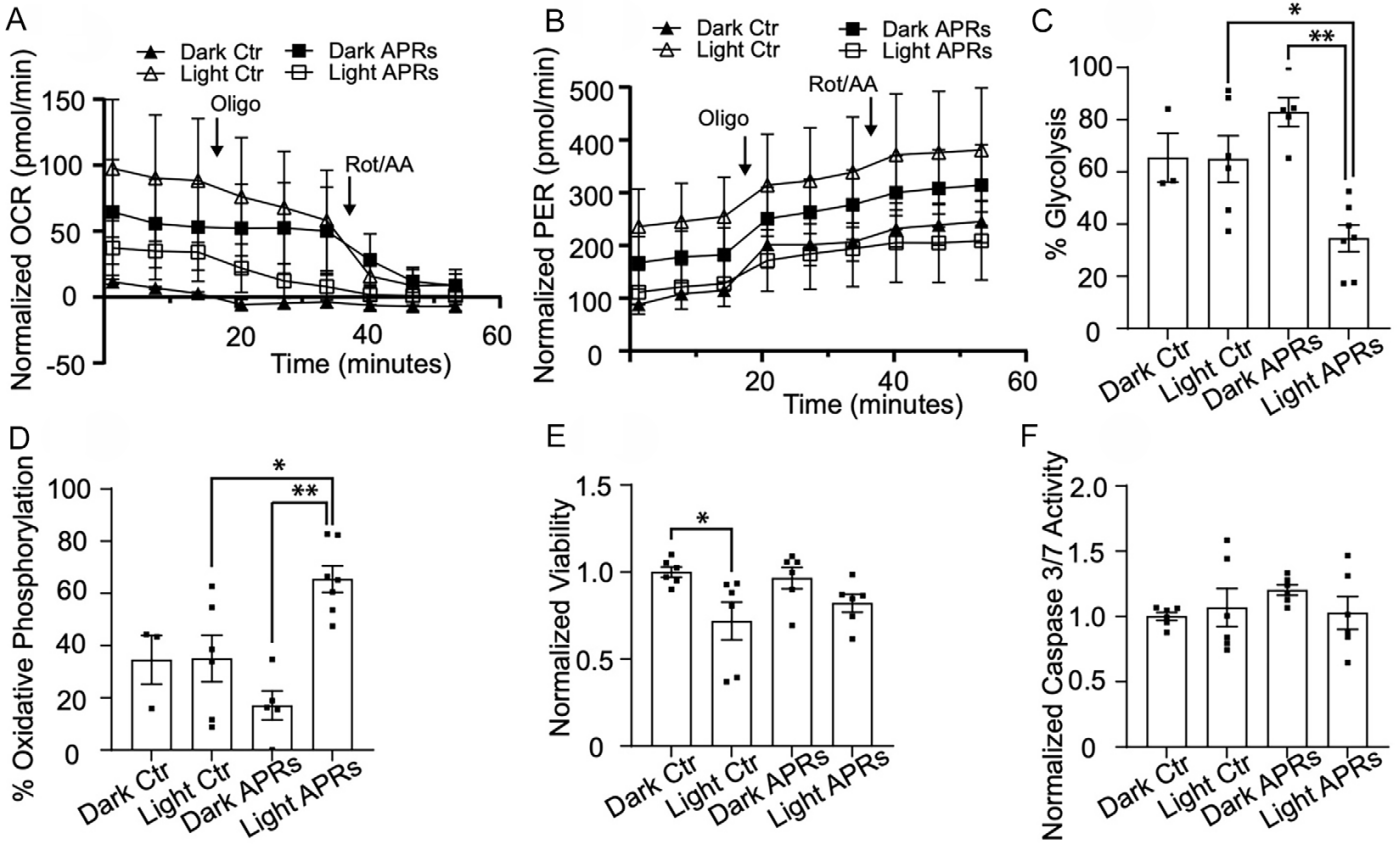
Measurements of cell area normalized (A) OCR and (B) PER from the Seahorse ATP rate assay, in hRGCs exposed to green light at 40 Hz for 24 h (light) or covered with foil (dark) with or without (control [Ctr]) 100 ppm APRs. Calculated (C) percent glycolysis, and (D) percent oxidative phosphorylation. *n* = 3–7 biological repeats. (E) Viability and (F) caspase 3/7 activity were measured using the ApoToxGlo‐Triplex assay kit in hRGCs with or without 100 ppm APRs, and exposure to green light at 40 Hz for 24 h. *n* = 6. *p < 0.05, **p < 0.01. Two‐way ANOVA with Tukey's post hoc test. All error bars are the SEM.

To validate the previous finding that hRGCs are light responsive in the presence of APRs, we also assessed their metabolic activity. RGCs are intrinsically reliant on oxygen‐dependent mitochondrial OXPHOS to maximize the ATP supply from a single glucose molecule for their high energy demands. It has been reported that in isolated mouse retinas, light flickering increases oxygen consumption in the inner retina, which contains RGCs, and that this effect is observed in healthy human subjects [45]. These indicate that under optical stimulation, RGCs may shift to more OXPHOS‐dependent metabolism. To obtain direct evidence of whether RGCs alter their metabolic status under optical stimulation through APR, we performed a Seahorse ATP rate assay on hRGCs under different conditions. In this assay, the instrument measures the oxygen and proton levels in the medium to extract the oxygen consumption rate (OCR) and proton efflux rate (PER) (Figure 4A,B) and subsequently calculates the percentages of glycolysis and OXPHOS. We observed that combined treatment with APRs and light reduced PER (Figure 4B) and, correspondingly, reduced the percentage of glycolysis (Figure 4C). Remarkably, light exposures in the presence of APRs significantly increased the percentage of OXPHOS in hRGCs, providing direct evidence that APRs can stimulate RGCs similar to photoreceptor cells (Figure 4D). For translational applications, we tested whether APRs are toxic to hRGCs with or without light stimulation. We observed a moderate decrease in cell viability when hRGCs were exposed to light alone (Figure 4E). However, in the presence of APRs alone or with light, no decrease in hRGC viability (Figure 4E) and no increase in apoptosis (Figure 4F) were observed, supporting its safe clinical application.

**FIGURE 5:**
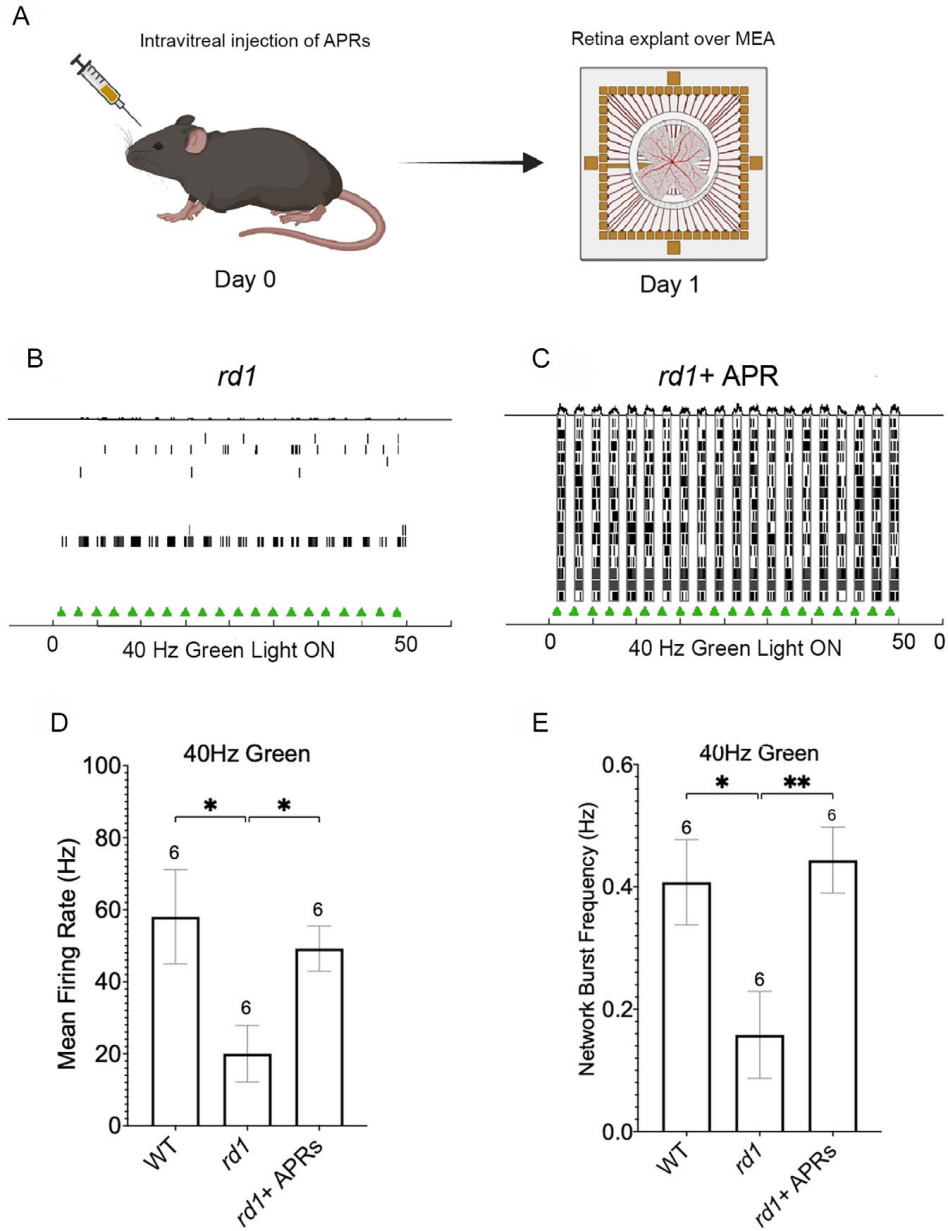
Depicts APR's function on explanted retinal tissue. APRs (1000 ppm/ml) were injected into *rd1* mice's vitreous at baseline, and 24 h later, the retina was separated and flattened on a MEA plate with the ganglion side toward recording electrodes (A). Optical stimulation, 1 s‐ON and 1 s‐OFF with 40 Hz green light from an LED source, was performed to measure the performance of APRs after IVI of APRs in the *rd1* mice retina explant. Raster plots in (B,C) show electrical activity recorded by MEA in *rd1* (sham‐treated) and *rd1* + APRs (IVI), respectively. Electrical responses improved in the retina explant of *rd1* + APR injection at both electrode (D) and network levels (E). Data are shown as mean ± SEM. Statistical comparisons were made using a mixed‐effects model with Dunnett's multiple comparisons test (sham‐treated *rd1* *n* = 6, WT *n* = 6, *rd1* + APRs *n* = 6). *p < 0.05, **p < 0.01.

Notably, for viability assays, hRGCs were exposed to 40 Hz green light at 100% LED intensity, corresponding to ≈1.9 mW/mm^2^ at the culture surface based on manufacturer specifications, an irradiance comparable to those used in retinal optical stimulation and MEA‐based studies [46]. Light exposure alone induced moderate hRGC activation (Figure 5F) and a modest increase in apoptosis in the absence of APRs (Figure 5E). In contrast, APR‐treated cultures exhibited enhanced light‐evoked firing without increased apoptosis or cell death under identical illumination conditions (Figure 5E,F), indicating that APR‐mediated optical stimulation does not compromise hRGC survival.

### 3.6 Intravitreal Artificial Photoreceptor Delivery Restores Retina Network Response in *rd1* Explants

To evaluate the efficacy of the APRs, visual responses were assessed 1 day after IVI in the *rd1* mouse model of retinal degeneration and compared with those of sham‐treated *rd1* mice, which received the same volume of 9% saline, and WT controls. Optical stimulation was delivered using the Lumos optical stimulation system (Axion BioSystems) with green (530 nm) and blue (475 nm) LEDs. The stimulation protocol consisted of alternating 1 s ON/1 s OFF light cycles delivered at a programmed LED intensity of 1% of the maximum calibrated output (1.9 mW/mm^2^) of the corresponding LED, as defined by the Axion Lumos system. Two stimulation frequencies were evaluated: 25 and 40 Hz. For the 25 Hz protocol, each stimulation cycle consisted of a 1‐ms light pulse, followed by a 39‐ms interval without illumination**.** For the 40 Hz protocol, each cycle consisted of a 1‐ms light pulse, followed by a 24‐ms interval without illumination.

### 3.7 Green Light

Table 1 illustrates a significant enhancement of the APR‐treated *rd1* retina response compared to the sham‐treated *rd1* under 25 Hz green light stimulation.

**TABLE 1:** MEA‐based retinal responses under 25 Hz green light stimulation.

Metric	Sham‐treated *rd1* (mean ± SEM)	WT (mean ± SEM)	*rd1 *+ APRs (mean ± SEM)	Improvement versus *rd1*, %
Total spikes (*n*)	246.7 ± 109.1	1001 ± 373.1	1741 ± 230.1	+605.7**
Mean firing rate (Hz)	9.17 ± 4.98	31.02 ± 13.1	53.74 ± 6.76	+486.0**
Active electrodes (*n*)	2.8 ± 2.8	7 ± 3.66	18.33 ± 1.25	+554.6***
Bursting electrodes (*n*)	2.0 ± 0.44	4.57 ± 2.05	9.46 ± 1.66 (13)	+373.1**
Network bursts (*n*)	2.8 ± 2.8	7 ± 3.66	18.33 ± 1.25	+554.6**
Network burst frequency (Hz)	0.0126 ± 0.0126	0.0954 ± 0.0372	0.193 ± 0.0185	+1434.2***

*Note* *:* Statistical comparisons were made using a mixed‐effects model with Dunnett's multiple comparisons test (*n* = 6).

** *p *< 0.01.

*** *p *< 0.001.

The enhancement is observed in total spikes and burst‐related metrics. The number of bursting electrodes increased significantly (sixfold). Network bursts increased 5.5‐fold, and, importantly, the network burst frequency rose 14‐fold and, in all cases, reached or exceeded the data recorded for WT.

Table 2 illustrates improvements in the APR‐treated *rd1* mouse compared to the sham‐treated *rd1* when 40 Hz green light stimulation is used. The level of excitation produced by the treatment of APRs resulted in a twofold increase in spikes. The number of bursting electrodes increased significantly (threefold). Network bursts increased 1.2‐fold; however, the network burst frequency rose 0.8‐fold. This was comparable to the data recorded for WT.

**TABLE 2:** MEA‐based retinal responses under 40 Hz green light stimulation.

Metric	Sham‐treated *rd1* (mean ± SEM)	WT (mean ± SEM)	*rd1* + APRs (mean ± SEM)	Improvement versus *rd1*, %
Total spikes (*n*)	560.8 ± 167.0	1860 ± 236.9	1786 ± 281.1	+218.5**
Mean firing rate (Hz)	20.01 ± 7.86	58.04 ± 13.05	49.20 ± 6.29	+145.9*
Active electrodes (*n*)	8.33 ± 3.77	14.5 ± 3.5	16.91 ± 1.51	+102.9*
Bursting electrodes (*n*)	5.5 ± 1.61	9.67 ± 2.95	9.18 ± 1.81	+66.9
Network bursts (*n*)	8.33 ± 3.77	14.5 ± 3.5	16.91 ± 1.51	+102.9**
Network burst frequency (Hz)	0.1425 ± 0.0713	0.2307 ± 0.0561	0.2631 ± 0.0341	+84.6**

*Note:* Statistical comparisons were made using a mixed‐effects model with Dunnett's multiple comparisons test (*n* = 6).

* *p *< 0.05.

** *p *< 0.01.

The summary of MEA parameters after excitation with 25 and 40 Hz green light in *rd1* mice 1 day after IVI of APRs, compared to sham‐treated *rd1* injection and wild‐type control, is depicted in Figure S3.

### 3.8 Blue Light

Table 3 illustrates a significant enhancement of the APR‐treated *rd1* retina response compared to the sham‐treated *rd1* under 25 Hz blue light stimulation. The enhancement is observed in total spikes and burst‐related metrics. The number of bursting electrodes increased significantly (twofold). Network bursts increased twofold, and, importantly, the network burst frequency rose 2.6‐fold and, in all cases, reached or exceeded the data recorded for WT.

**TABLE 3:** MEA‐derived retinal responses under 25 Hz blue light stimulation.

Parameter	Sham‐treated *rd1* (mean ± SEM)	WT (mean ± SEM)	*rd1* + APRs (mean ± SEM)	Improvement versus *rd1*, %
Spikes (count)	1295 ± 314	4786 ± 160	3324 ± 336	+156.7**
Mean firing rate (Hz)	20.0 ± 4.93	71.43 ± 4.48	53.82 ± 7.76	+169.1**
Active electrodes (*n*)	13.88 ± 0.61	16 ± 0	16 ± 0	+15.3***
Bursting electrodes (*n*)	3.13 ± 1.04	15.63 ± 0.26	10.20 ± 1.97	+226.4**
Network bursts (*n*)	4.63 ± 3.03	20.38 ± 0.18	15.20 ± 2.67	+228.6**
Network burst frequency (Hz)	0.116 ± 0.076	0.509 ± 0.005	0.422 ± 0.058	+265.2**

*Note:* Statistical comparisons were made using a mixed‐effects model with Dunnett's multiple comparisons test (sham‐treated *rd1 n* = 8, WT *n* = 8, *rd1* + APRs *n* = 10).

** *p *< 0.01.

*** *p *< 0.001.

Table 4 illustrates a significant enhancement of the APR‐treated *rd1* retina response compared to the sham‐treated *rd1* under 40 Hz blue light stimulation. The enhancement is observed in total spikes and burst‐related metrics. The number of bursting electrodes increased slightly (1.5‐fold). Network bursts increased 2.7‐fold, and, importantly, the network burst frequency rose 2.7‐fold and, in all cases, reached or exceeded the data recorded for WT.

**TABLE 4:** MEA‐derived retinal responses under 40 Hz blue light stimulation.

Parameter	Sham‐treated *rd1* (mean ± SEM)	WT (mean ± SEM)	*rd1* + APRs (mean ± SEM)	Improvement versus *rd1*, %
Spikes (count)	800.1 ± 197	2857 ± 179	2153 ± 310	+169.1***
Mean firing rate (Hz)	32.37 ± 7.84	119.7 ± 4.00	83.10 ± 8.39	+156.7***
Active electrodes (*n*)	14.75 ± 0.3134	16 ± 0	16 ± 0	+8.5***
Bursting electrodes (*n*)	5.50 ± 1.97	16 ± 0	14.20 ± 0.83	+158.2***
Network bursts (*n*)	5.38 ± 3.20	20 ± 0	20 ± 0	+272.1***
Network burst frequency (Hz)	0.134 ± 0.080	0.50 ± 0	0.50 ± 0	+272.0***

*Note:* Statistical comparisons were made using a mixed‐effects model with Dunnett's multiple comparisons test (sham‐treated *rd1 n* = 8, WT *n* = 8, *rd1* + APRs *n* = 10).

*** *p *< 0.001.

Detailed MEA parameters for 25 and 40 Hz blue light in *rd1* mice with IVI of APRs, compared to *rd1* w/o injection and wild‐type control, are shown in Figure S4.

Raster plots for different frequency‐color optical stimulation show how IVI‐APRs improve both electrode and network activity in the *rd1* mice retina explant (Figure S5).

### 3.9 In Vivo Readouts Confirm Transient Functional Restoration After Artificial Photoreceptor Injection Pupillary Light Reflex

**FIGURE 6:**
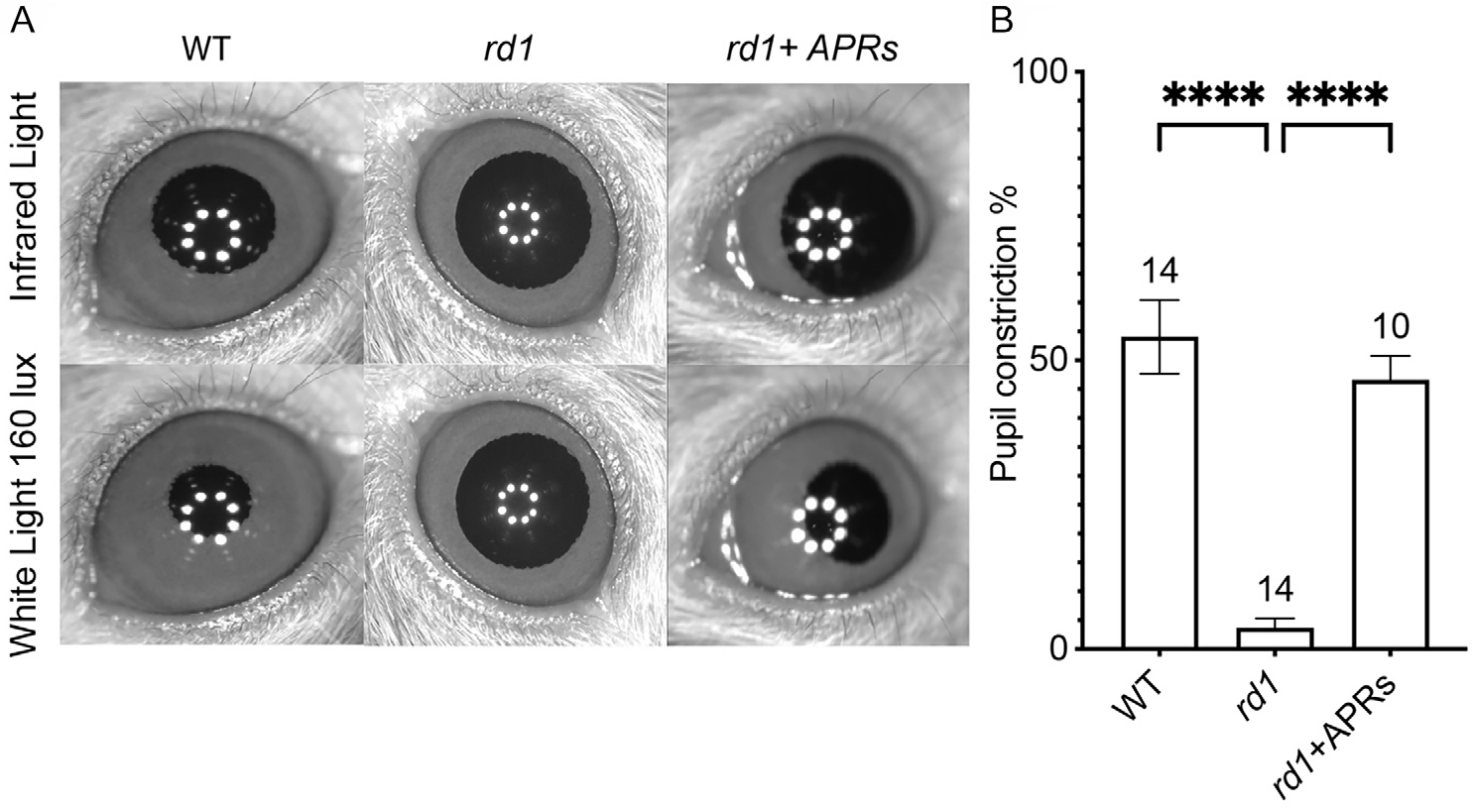
PLR in WT vs. *rd1* (blind) mice and with APR. APRs were injected into the vitreous of *rd1* (blind) mice and evaluated using the PLR. Panel (A) shows a pupillary constriction in *rd1* + APRs exposed to white light. The constriction of the pupil (%) following light exposure is much less in blind mice and can be significantly rescued within 1 day following IVI of the APR (B). Data are shown as mean ± SEM. Statistical comparisons were made using the Kruskal–Wallis test with Dunnett's multiple comparisons. ****p < 0.0001.

The results of PLR (area of pupil) 1 day after IVI‐APRs, reflected in Figure 6, revealed significantly increased pupillary constriction in *rd1* + APRs (46.64 ± 4.11, *p *< 0.0001) in response to white light compared to *rd1* without IVI‐APRs (3.7 ± 1.66).

### 3.10 Visual Evoked Potential

According to the protocol described in the method section, MEA results showed an increased N1 amplitude on Day 1 after APR injection into the *rd1* vitreous, followed by a gradual decrease as the APRs were washed out of the vitreous. For green light stimulation, the N1 amplitude on Day 1 after injection was statistically higher than the preinjection day (−16.32 vs. −33.10, *p *= 0.003), and this difference remained until Day 3 as well (−16.32 vs. −27.17, *p *= 0.038).

**FIGURE 7:**
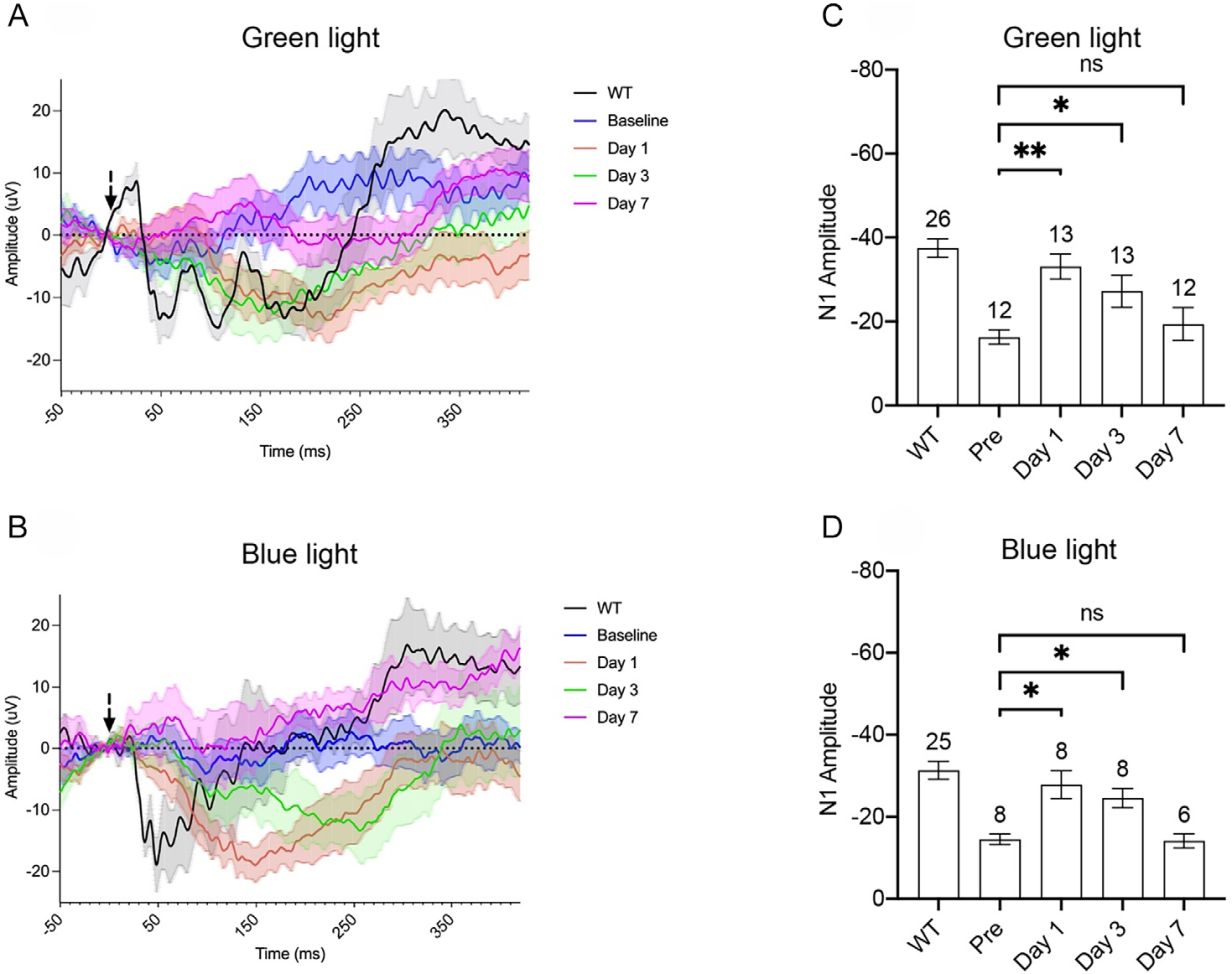
Shows VEP activity of *rd1* mice that have received IVI‐APRs injection (*rd1* + APRs), compared to *rd1* and WT mice over 1 week from preinjection timepoint for green (A) and blue (B) light. N1 amplitude increased significantly in the Day 1 measurement and remained significantly higher on Day 3 post‐IVI for both green (C) and blue (D) optical stimulation. However, the change in N1 amplitude did not last on Day 7 of the post‐IVI VEP test. Data are shown as mean ± SEM. Statistical comparisons were made using a mixed‐effects model with Dunnett's multiple comparisons test. *p < 0.05, **p < 0.01.

We observed similar patterns in the blue optical stimulation: N1 amplitude increased significantly on the day after APR injection compared to the preinjection day (−14.55 vs. −27.87, *p *= 0.036). It remained significant on Day 3 after injection (−14.55 vs. −24.6, *p *= 0.032). Figure 7A,B shows the average VEPs of WT and *rd1* mice receiving intravitreal APR injection over time, compared to their baseline for green and blue light stimulation, respectively. We observed a late‐phase negative VEP after light stimulation (arrow), which generally had a higher negative amplitude on Day 1 and Day 3 postinjection than at baseline or on Day 7. This finding is consistent with our observation that, using OCT, APRs are visible in the vitreous until Day 7 postinjection. The amplitude of N1, measured by the machine's built‐in algorithm, was statistically higher on Day 1 and Day 3 post‐IVI‐APR injection compared to the baseline and Day 7 for green and blue light stimulation (Figure 7C,D, respectively).

The N1 amplitude change on Day 1 after APRs injection was higher in green light stimulation compared to the blue, but was not statistically significant (16.78 vs. 13.32, *p *= 0.28).

## 4 Discussion

APRs were developed as injectable colloidal nanoparticle composite solutions. The structure consisted of a metallic core nanoparticle coated with a dielectric layer and mounted on a stronger dielectric core (Figure 1). These APRs demonstrated effective visible light‐to‐voltage transduction with enhanced polarity, compatible with RGC physiological stimulation.

A multiscale validation across hRGC cultures, *rd1* retina explants, and in vivo behavioral analyses, such as PLR and VEP, demonstrated that: 1) APRs effectively interfaced with the nerve cells and act as a photodetector; 2) APRs could drive cell‐level on‐demand activations and generated functional outcomes. The observed OXPHOS shift provides evidence of activation beyond electrophysiology and suggests that APR‐mediated stimulation resulted in characteristics consistent with those of highly active RGCs. It provided direct metabolic evidence that RGCs shift to mitochondrial OXPHOS under optical stimulation, which had only been inferred from indirect ex vivo and human subject studies [45, 47]. APRs did not induce apoptosis under test conditions. A metabolic change occurred only in the presence of APR and light without any cellular toxicity. This suggests that APRs could safely stimulate hRGCs as a potential photoreceptor prosthetic.

Multiple MEA experiments under green (530 nm) and blue (475 nm) optical stimulation demonstrated that APR treatment significantly improved electrophysiological activity in both cultured hRGCs and *rd1* retinal explants. Overall, blue light stimulation elicited higher absolute spiking and network activity across conditions (Figures S3–S5). However, APR‐treated *rd1* explants exhibited greater relative rescue under green illumination, where several MEA parameters consistently matched or exceeded WT levels. This suggests enhanced spectral coupling between the APRs and the retina at green wavelengths. This wavelength dependence is consistent with the UV–Vis absorbance maximum of the APRs at 520–525 nm (Figure 1D).

Following IVI of APRs into *rd1* mice, VEP recordings showed a significant increase in N1 amplitude at Day 1 postinjection relative to baseline, which then declined over subsequent days and was no longer significant by Day 7. The temporal profile confirmed the OCT findings in a subset of eyes, in which APRs were apparent in the vitreous and near the inner retinal surface on Day 1 and progressively redistributed or diminished by Day 7.

On Day 1, green stimulation elicited a larger increase in N1 amplitude than blue (ΔN1: 16.78 vs. 13.32 μV); however, this difference did not reach statistical significance, consistent with the limited sample size. Independent evidence of functional engagement of inner retinal pathways was provided by the PLR, which improved significantly in mice that received intravitreal APRs compared with their preinjection baseline.

## 5 Conclusion

Dystrophic and degenerative retinal disorders, including AMD and RP, are among the leading causes of photoreceptor loss, affecting 200 million and 1.5 million people worldwide, respectively [48, 49]. In many retinal conditions, despite extensive photoreceptor loss, inner layers might remain unaffected and preserve their functions. Therefore, treatment paradigms for these disorders would include preventing photoreceptor loss, regenerating photoreceptors, or shifting the treatment target to the inner retinal layers, thereby bypassing damaged photoreceptor layers.

Despite advances in the treatment of AMD, particularly exudative type, including antiangiogenic agents and photodynamic therapy for some subtypes [50, 51, 52], and ongoing research for the treatment of RP including gene delivery, neuroprosthetic retinal implants, optogenetic therapy, and injection of human ciliary neurotrophic factor (CNTF), the photoreceptor loss remains a challenging area without effective treatment [53, 54].

Electrical stimulation or optical induction of the RGCs has been used to bypass damaged photoreceptors and restore vision, which can be done via direct electrical stimulation, prostheses, or light‐based activation, such as amino acids and proteins for light‐gated ion channels, or in our case, nanoparticles [55, 56, 57]. The FDA‐approved bioelectrical implant declined in popularity in clinical settings due to the device's complexity, low image resolution, surgical complexity, potential side effects, and financial issues. Likewise, optogenetic therapy results, which involve transfection of ganglion cells with light‐sensitive opsins via viral vectors, thereby making them APRs, remain unclear and are currently not applicable in clinical settings [58].

In this study, we utilized nanocomposites that provide a new avenue for less complex treatment that does not rely on extensive surgery, electronic devices, or external optical systems. Compared with currently available retinal prostheses, including the Argus II, Alpha AMS, and PRIMA systems, which require complex vitreoretinal surgery and implantation of rigid electronic hardware, the injectable APR platform offers a minimally invasive alternative that can be delivered through IVI. Existing retinal implants are associated with surgical complexity, dependence on implanted electronics and, in some systems, external cameras and image‐processing units. In addition, the rigid structure of these implants may not fully conform to the curved retinal surface, potentially resulting in mechanical mismatch, suboptimal electrode–retina coupling, and device‐related complications such as fibrosis, retinal detachment, conjunctival erosion, infection, or device failure. Furthermore, the visual resolution of current prostheses is inherently limited by electrode density. In contrast, APRs directly convert incident light into local electrical stimulation without implanted electronics or external power sources [59, 60, 61]. Owing to their nanoscale size and injectable nature, APRs have the potential to achieve improved conformity with retinal tissue, broader retinal coverage, and reduced procedural complexity. Nevertheless, further studies are required to establish their long‐term biodistribution, safety, biocompatibility, and functional durability before clinical translation.

Targeting RGC activation is an important step to ensure the efficiency and safety of APRs. We developed injectable APRs that show potential to generate electrical fields in response to simulations of different wavelengths and frequencies of light. When gold nanoparticles are irradiated at a specific wavelength in the electromagnetic spectrum, the electrons around the particles begin to oscillate, producing an electric field known as LSPR. We implemented this phenomenon to produce electrical signals in response to light stimulation. In addition, we utilized PVDF, an FDA‐approved biocompatible polymer, to cover AuNPs, forming a nanocomposite, and mounted the nanocomposites in the core of Barium titanate to enhance the dielectric properties of the particles, thereby improving their capability to deliver electrical current efficiently into ganglion cells. Other advantages of the product include sensitivity to different frequencies, intensities, and durations of stimulation, which give it the potential to produce high‐quality natural vision. Additionally, these APRs can detect different visible light spectrums, yielding potential for color vision.

This study was a collaborative project among clinical, basic science, and engineering departments, with expert specialists across various domains. We assessed the potential of the APRs through various in vivo and in vitro experiments, which yielded robust results. However, some limitations need to be addressed in the future to pave the way for the application of APRs in clinical settings.

Based on OCT imaging, the APRs disappeared significantly after day seven, indicating a short half‐life, and the electrophysiological study with VEP confirmed this by demonstrating decreased N1 amplitude over time. This is a major drawback that requires frequent injections to maintain viable electrical activity. We did not investigate the intraocular distribution, retinal localization, or clearance of APRs following IVI; therefore, the final fate and biodistribution of the nanoparticles within the eye remain to be determined. Second, because the residence time of APRs in the vitreous is limited, the present study was not designed to evaluate their long‐term efficacy or safety and evaluation of potential long‐term complications like chronic inflammation, epiretinal membrane formation, retinal vascular alterations, changes in intraocular pressure, and vitreous opacities. Future studies incorporating prolonged follow‐up, biodistribution analyses, and histological assessment will be necessary to establish the long‐term biocompatibility and translational potential of this platform.

In addition, functional efficacy was evaluated using electrophysiological (VEP) and reflexive (PLR) assessments, which demonstrate restoration of light‐evoked retinal and visual pathway activity but do not directly establish recovery of higher‐order visual function. Although these findings provide important proof‐of‐concept evidence for the ability of APRs to restore light responsiveness, additional behavioral assessments, including optomotor response, visual cliff, and pattern discrimination tests, will be required to determine the extent of functional vision restoration preferably in a larger animal model.

Another limitation for all interventions like ours that bypass photoreceptors by direct activation of RGCs is that the activation of RGCs is not selective and specific, which means all RGC types may be activated at the same time by the same stimulation. Not surprisingly, the shape of the average VEP curves in *rd1* + APRs is not exactly similar to the average WT–VEP curve (Figure 5A,B), and the amplitude and latency of VEP parameters could further be affected by the plasticity of the visual cortex in *rd1* mice with already‐degenerated photoreceptors from the earliest hours of their life. Additionally, the stimulation profile in terms of light intensity, frequency, and familiarity of subjects with stimulation could affect the VEP shape.

The stability of the nanoparticles and their attachment to the target, in this case, RGCs, is another critical factor in developing APRs.

Therefore, future applications could include slow‐release capsules, such as poly (lactic‐co‐glycolic acid) (PLGA), to deliver APR nanoparticle composites to the desired area to prolong treatment without frequent injections [62, 63, 64, 65, 66]. Other possibilities include altering the nanoparticle metal, stabilizer, dielectric polymer, or dielectric core to better transfer the LSPR wavelength electron oscillating current to the cells, make it more stable and viable for patients, and improve the recovery of sight [67, 68, 69, 70, 71, 72]. It may be possible to regain sight of different colors, frequencies, and intensities of light, and at a higher level of recovery [73, 74]. Methods including a linker molecule or hydrogels like polyethylene glycol (PEG), peptides such as arginyl glycyl aspartic acid (RGD), phase‐shifting polymers like poly (*N*‐isopropyl acrylamide) (pNIPAM), and the previously mentioned slow release, as with PLGA are all potential avenues of improving this treatment half‐life and attachment [75, 76, 77].

In addition, future investigation into making retinal implants with APRs on the surface using precise methods, such as two‐photon lithography, would be an alternative to IVI injection of APRs. More experiments are needed to further demonstrate the potential of nanocomposites to optically evoke RGCs in place of damaged or absent photoreceptors. However, this is a promising approach alongside other existing and developing methods.

## Funding

The study was supported by Research to Prevent Blindness, NEI R2|EY032652, and Falk Medical Trust.

## Conflicts of Interest

The authors declare no conflicts of interest.

## Supporting information

Supplementary Material

## Data Availability

The data supporting the findings of this study are available from the corresponding author upon reasonable request.
