# Supplementary material for: Injectable Artificial Photoreceptors: STEM Cell Functional Integration and in vivo Electrophysiological Validation in Retinal Degeneration Models
Source: Small Sci. 2026 Jul 31;6(8):e70353. doi: 10.1002/smsc.70353 (PMC13431967; doi:10.1002/smsc.70353)
Supplement: Supplementary file 1 — Supplementary Material [file SMSC-6-e70353-s001.pdf]

Retinal explant Isolation

Following anaesthesia, the eyeballs were enucleated using forceps and immediately transferred to oxygenated Ames' medium (A1420, *Sigma-Aldrich*) at 33 °C, with pH maintained at 7.4 by continuous bubbling of a 95% O_2 / 5% CO_2 gas mixture.

To prevent photoreceptor bleaching, all subsequent dissection steps were performed under red-light illumination using a laboratory microscope. A corneal incision was initiated with a 28-gauge needle and extended to the limbus using fine spring scissors. The anterior segment was removed via a 360° circumferential incision, followed by gentle extraction of the crystalline lens to minimize the risk of retinal tissue displacement.

Using fine forceps and scissors, retinal adhesions at the anterior margin and optic disc were carefully released. Residual vitreous was delicately removed from the retinal surface to ensure optimal contact with the microelectrode array (MEA). The isolated retina was then flattened by making four precise radial incisions and mounted ganglion cell side up on a custom-made permeable mesh.

The prepared retinal tissue was then inverted (ganglion cell side down) onto the recording well of the MEA system, ensuring firm contact with the electrodes. Oxygenated Ames' medium was added to the well to sustain physiological conditions for the ganglion cells, while pH and temperature were continuously regulated by a built-in CO_2 convection system.

This preparation procedure was performed for retinas harvested from ***rd1***, ***rd1* + APR**, and **wild-type** mice.

Microelectrode array (MEA)

Micro electrode array (MEA; Maestro Edge, Axion Biosystems, Atlanta, GA) was used to record biologic electrical signals in both human stem cell derived cultured ganglion cells and retina explant using 96 well plate (Axion Biosystems) and 24-well plate respectively.

Light stimulation was performed by Lumos optical stimulation module (Axion BioSystems) for green (530nm) and blue (475nm) in different frequencies in a pattern of light on-off cycles.

Recording of the signals was performed by AxIS Navigator software (version 3.5.2; Axion BioSystems, Atlanta, GA, USA).

Spike detection cutoff assigned between 5.75 -7 SD of amplitudes, depending on the background noises. MEA parameters including number of spikes, mean firing rate, number of active electrodes, number of bursting electrodes, number of network bursts and network burst frequency were extracted using Neural Metric Tools (Axion Biosystem) for each well (treatment group) and analysed. Following is the definition and criteria for the parameters:

Number of spikes: total number of spikes that have higher amplitude than the predefined threshold in SD during stimulation time.

Mean firing rate (Hz)=

[illegible] *Number ofspikesstimulation times s*

Number of active electrodes: number of electrodes that shown at least 5 spikes/minute.

Number of bursting electrodes: Inter-spike interval (ISI) method was utilized with at least 5 spikes and maximum ISI of 100ms.

Network burst frequency: ISI method was utilized with at least 50 spikes, maximum 100ms for ISI and at least involving 35% of electrodes.

Visual evoked potential (VEP)

Mice were anesthetized for 1h with 5-10mg/kg Xylazine or 0.5mg/kg Dexmedetomidine and 75mg/kg Ketamine via intraperitoneal injection.

Celeris ([Diagnosys LLC]), was used for VEP recording contact electrodes to the cornea with normal saline as interfacial fluid to improve conduction. Reference and ground electrodes were inserted as follow: one needle electrode at the tail base, one at the mouth, and one at the occiput (midline between the ears)

We implemented built-in default flash VEP protocols for VEP measurement. Since APRs were more sensitive to the green and blue light, the LED light source set as green and blue light.

The electrical activity was recorded from 50ms before the flashlight and 420ms afterward. Sampling rate was 2000Hz and number of sweeps set as 100 times. Recoding was performed using the Epsion software (Diagnosys) averaging all sweeps. The built-in algorithms were used to detect and measure N1 wave amplitude.

Supplementary figures

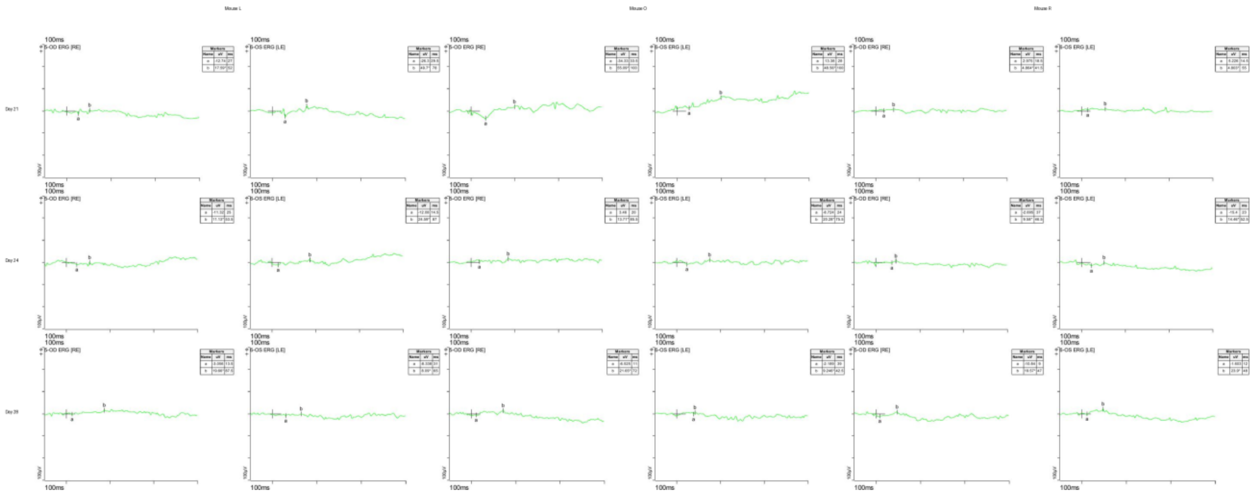

Figure 1. Serial ERG recordings from *rd1* mice demonstrate progressive retinal degeneration, resulting in absent or markedly attenuated retinal electrical responses.

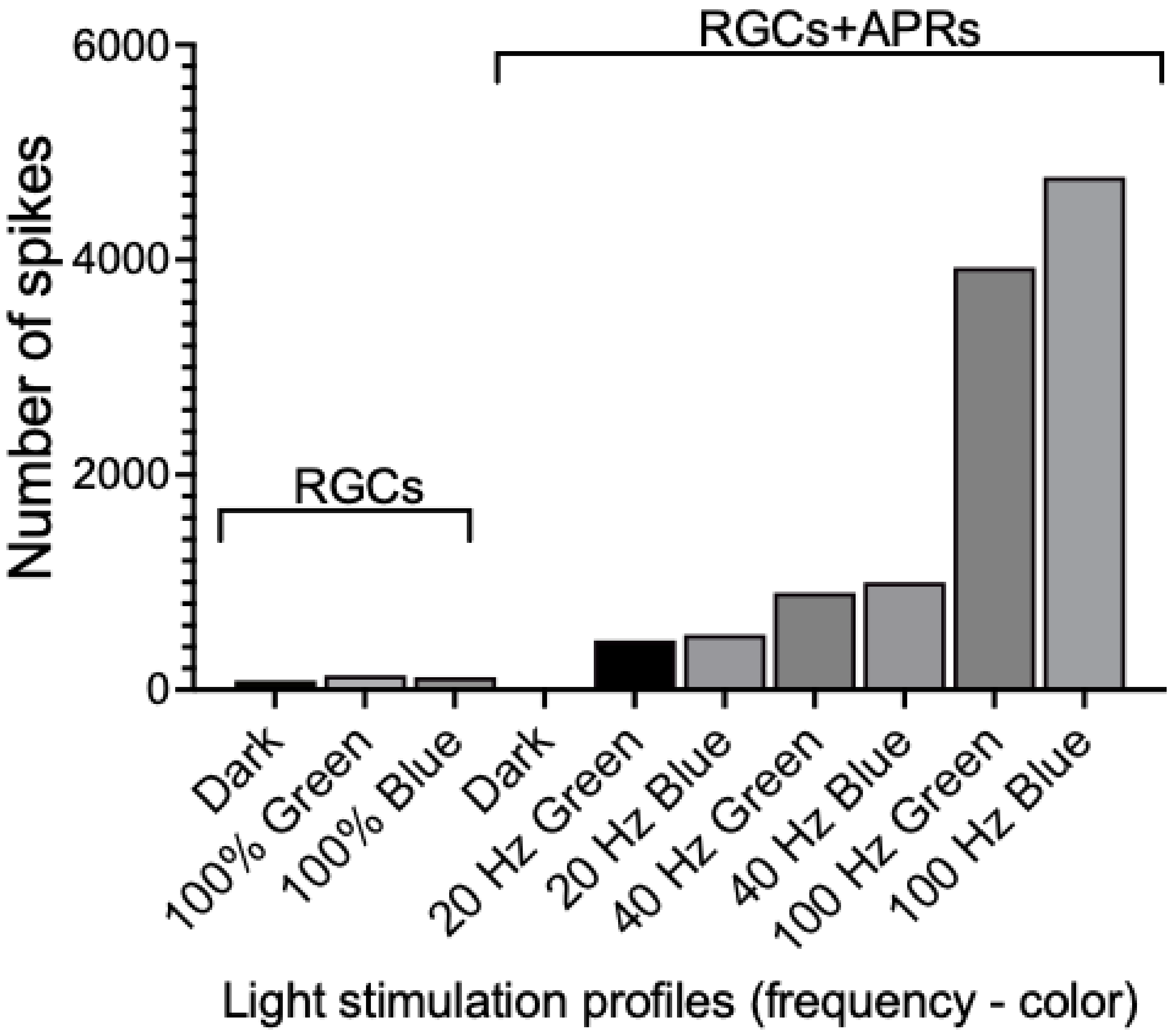

Figure 2. APRs function in different colors and frequencies. Cultured RGCs in MEA plates showed increased number of spiks by increasing the frequency of optical stimulation in presence of 100ppm APRs for both green and blue spectrum.

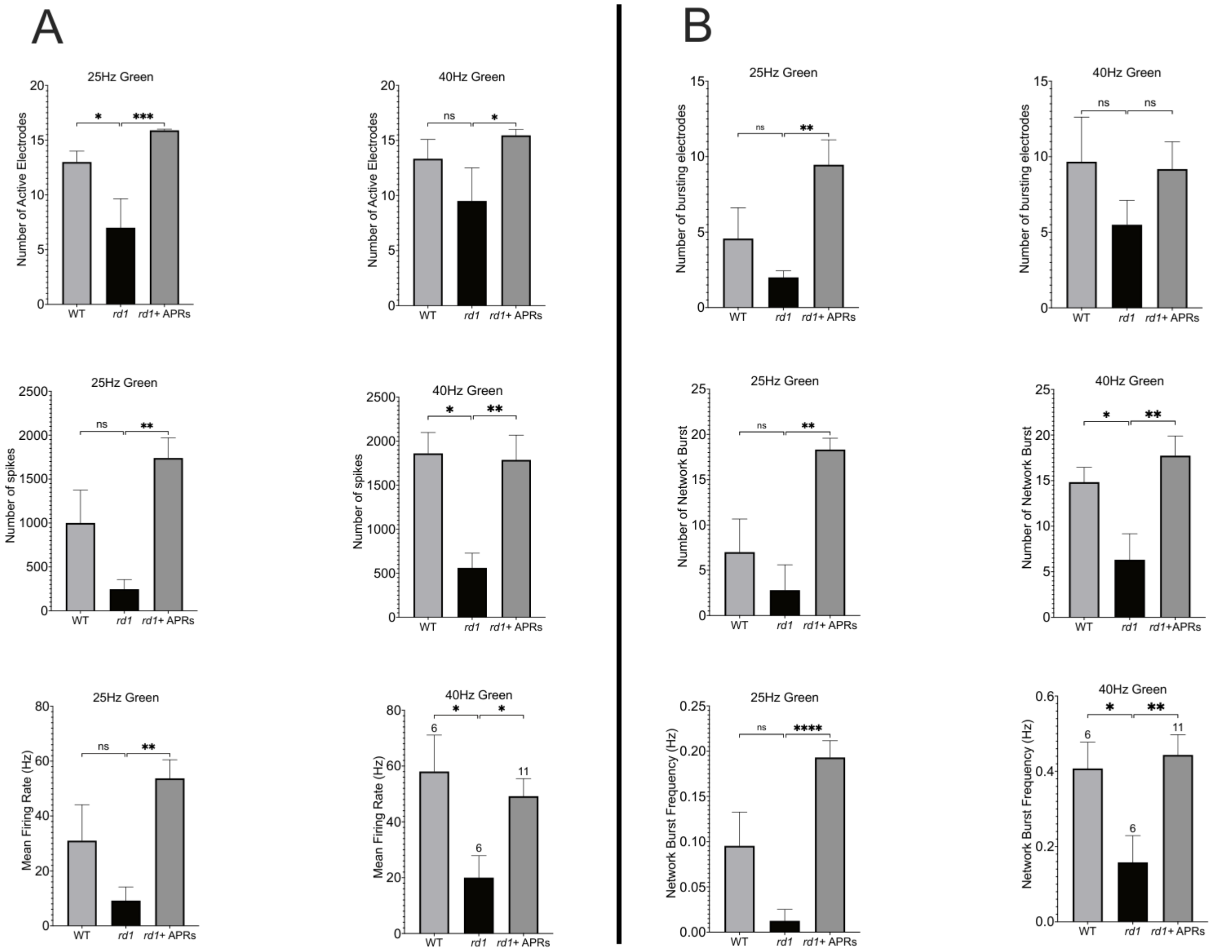

Figure 3. Comparison of electrode activity **(A)** and burst parameters **(B)** of *rd1* retinal explants without APRs, *rd1* retinal explants one day after intravitreal APRs injection (*rd1*+APRs), and wild type (WT), with 1s on-1s off green light stimulation at different frequencies of 25Hz and 40Hz, using Micro Electrode Array (MEA).

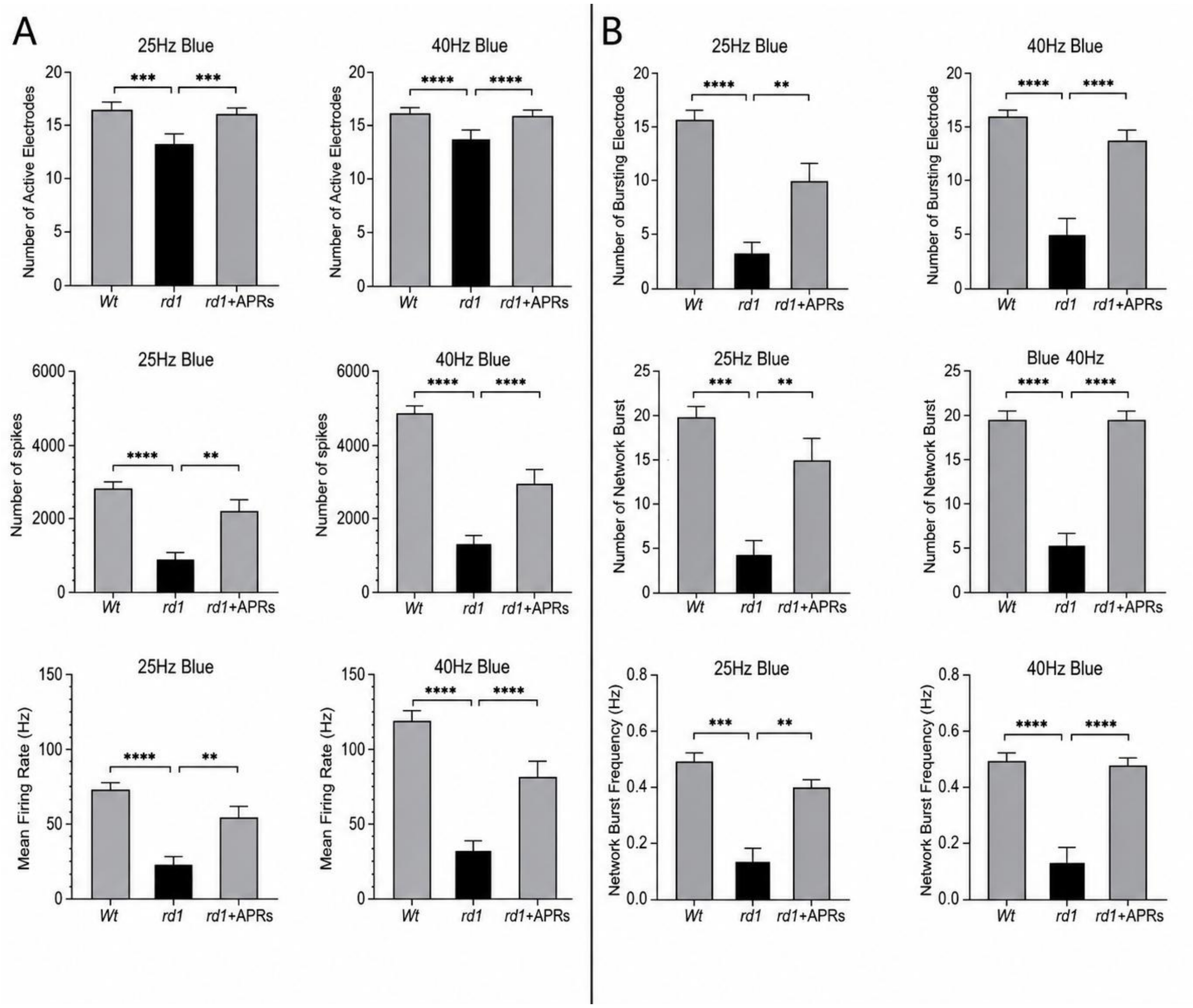

Figure 4. Comparison of electrode activity (A) and burst parameters **(B)** of *rd1* retinal explants without APRs, *rd1 retina* one day after intravitreal APRs injection (*rd1*+APRs) and, wild type (WT) with 1s on-1s off blue light stimulation in different frequency of 25Hz and 40Hz, using Micro Electrode Array (MEA).

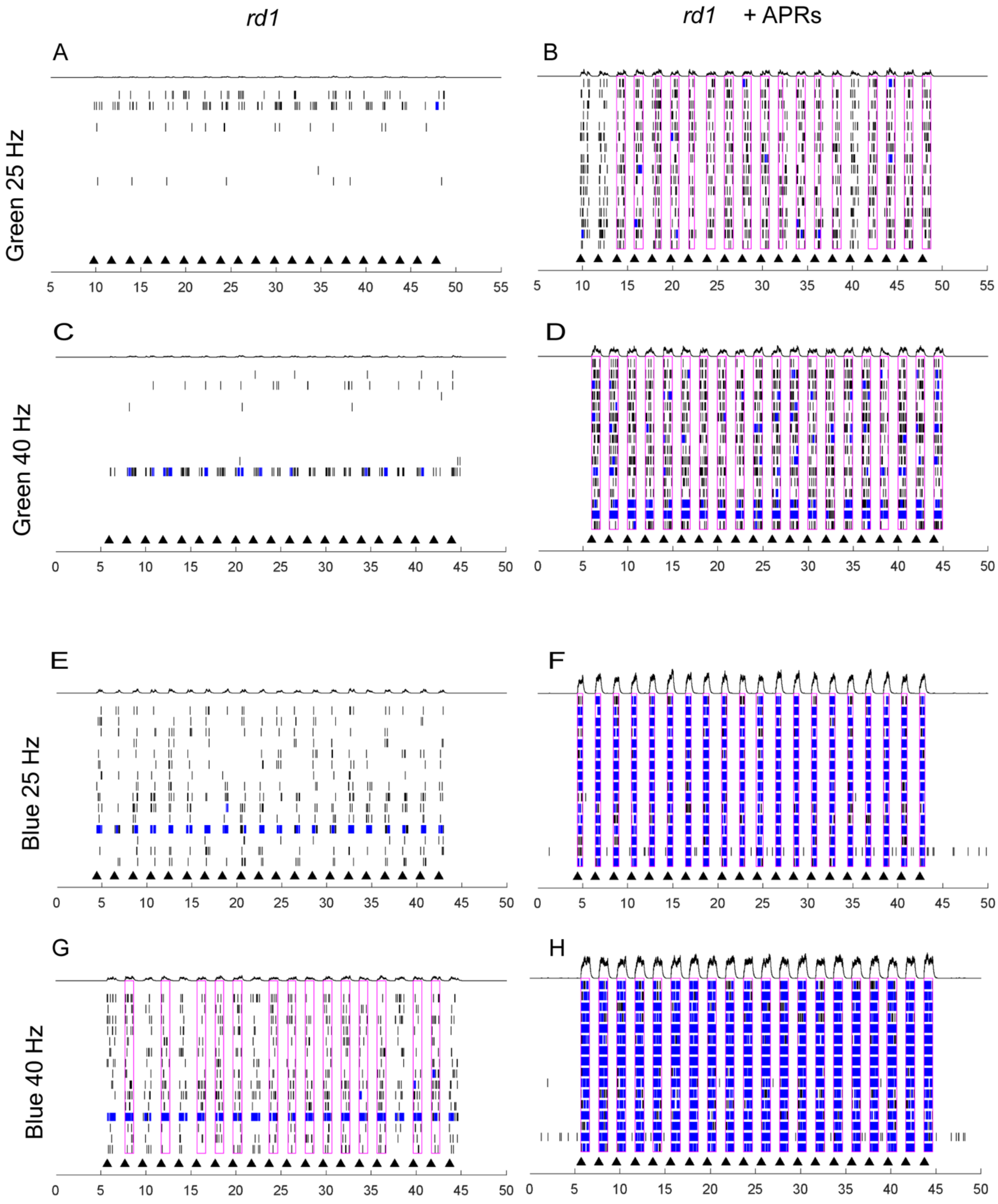

Figure 5. Demonstration of raster plots in different colour-frequency optical stimulation in *rd1* retina explant treated with APRs in in comparison to none treated *rd1* retina explant.
